# Familial resemblance and family-based heritability of nutrients intake in Iranian population: Tehran cardiometabolic genetic study

**DOI:** 10.1186/s12889-023-16708-2

**Published:** 2023-09-14

**Authors:** Farshad Teymoori, Mahdi Akbarzadeh, Hossein Farhadnejad, Parisa Riahi, Ebrahim Mokhtari, Hamid Ahmadirad, Asiyeh Sadat Zahedi, Firoozeh Hosseini-Esfahani, Maryam Zarkesh, Maryam S. Daneshpour, Parvin Mirmiran, Mohammadreza Vafa

**Affiliations:** 1 Department of Nutrition, School of Public Health, Iran University of Medical Sciences, Tehran, Iran; 2 Cellular and Molecular Endocrine Research Center, Research Institute for Endocrine Sciences, Shahid Beheshti University of Medical Sciences, Tehran, Iran; 3 Nutrition and Endocrine Research Center, Research Institute for Endocrine Sciences, Shahid Beheshti University of Medical Sciences, Tehran, Iran; 4 Department of Community Nutrition, School of Nutrition and Food Sciences, Shiraz University of Medical Sciences, Shiraz, Iran

**Keywords:** Nutrients, Macronutrients, Micronutrients, Heritability, Familial resemblance, Genetics, Environmental factors

## Abstract

**Background:**

We aimed to investigate the familial resemblance of dietary intakes, including energy and nutrients, and the family-based heritability of dietary intake in different age-sex dyads of the Tehran cardiometabolic genetic study.

**Methods:**

This cross-sectional study was conducted on 9,798 participants, aged ≥ 18 years, with complete data in each of the third, fourth, fifth, and sixth surveys of the Tehran Cardiometabolic Genetic study, who were eligible to enter the current study based on inclusion and exclusion criteria. Nutrient intake was determined using a valid and reliable food frequency questionnaire (FFQ). FCOR command of the S.A.G.E. software was used to estimate the intra-class correlation coefficients of all relative pairs to verify the family resemblance of dietary nutrient intakes. Classical likelihood-based is used to assess the family-based heritability of dietary nutrient traits.

**Results:**

There were 4338 families with a mean family size of 3.20 ± 2.89, including 1 to 32 members (2567 constituent pedigrees and 1572 singletons) and 3627 sibships. The mean ± SD age of participants was 42.0 ± 15.2 years, and 44.5% were males. The heritability of nutrient intake ranged from 3 to 21%. The resemblance degree of energy intake and most nutrients between spouses or between parents and children is weak to moderate; however, a high resemblance of intake was observed for some food components, especially among spouses, including trans fatty acids (TFAs) (r:0.70), chromium (r:0.44), fiber(r:0.35), pantothenic acid (r:0.31), and vitamin C(r:0.31). Based on our findings, the resemblance of nutrient intake in spouses was greater than in parent-offspring. The similarity in parent–offspring nutrient intake was different, and the correlation in mother-girls nutrient intakes was greater than other parent–child correlations. Also, the lowest resemblance in nutrient intake was observed among siblings.

**Conclusions:**

Our findings suggested a weak-to-moderate similarity between the nutrient intakes of parents and offspring. The resemblance degree in nutrient intake varied between different family pairs; the strongest correlation of nutrients was observed between spouses, which includes TFAs, chromium, fiber, pantothenic acid, and vitamin C. The lowest correlation of nutrients was between siblings, such as carbohydrates, thiamine, niacin, and vitamin K. An individual's nutrient intake can somewhat be influenced by genetics, family relationships, and the effects of parents, although the significant influence of environmental factors should not be ignored.

**Supplementary Information:**

The online version contains supplementary material available at 10.1186/s12889-023-16708-2.

## Introduction

In the past, the similarity of nutritional traits, such as food intake between family members, has attracted the attention of researchers worldwide. It seems that family environment and genetics have the biggest role in this similarity, at least in the early years of life [1]. Nowadays, it is accepted that nutritional traits such as dietary intake are heritable [2]. Heritability determines the share of genetics and environment in the variation of a trait in the population [3]. Twin and family-based studies are the main studies investigating traits' heritability. Most of the available studies in this field have been conducted on twins.

Twin studies estimate heritability for total energy intake ranging from 38 to 51%. This estimate in family studies is from 12 to 54%. Heritability of macronutrient intakes is estimated at 1–58% in twins and 12–61% in family studies [2–13]. However, information on the heritability of intake of other nutrients, minerals, electrolytes, and the sources of macronutrients, is limited. In a study by Cai et al., the heritability of polyunsaturated fatty acids (PUFAs), monounsaturated fatty acids (MUFAs), and saturated fatty acids (SFAs) was reported to be 0.09, 0.21, and 0.2, respectively [3]. Lieske et al. estimated the heritability of calcium, oxalate, fructose, and sucrose as 0.56, 0.25, 0.26, and 0.37, respectively [10]. In most studies, the heritability of macronutrients is reported in one of the two scales, grams per day or percentage of energy, and studies that report both modes are few.

From another view, the resemblance of dietary intakes among parents and offspring has already been investigated worldwide [1, 14]. This phenomenon is based on the fact that parents provide their children's genes and home environment and therefore have a key role in shaping children's early experiences with food and eating behaviors. Family members share many traits that make them the appropriate candidates to reveal the effects of genes and the environment on dietary intake. For example, types of food served at home or outside, cooking methods, frequency of meals, decisions about where the family eats out, and generally how a child thinks about food are all environmental-related factors determined by parents [15].

However, most previous studies showed a weak to moderate correlation between parents' and offsprings' dietary intakes [1, 16]. This may be because young individuals eating patterns are affected by many complex factors, and in a comprehensive view, the family environment, with all its importance, is placed next to other social and biological factors affecting dietary intake. Also, as children grow older, the family's influence on food choices gradually diminishes [17]. Furthermore, most studies investigating the resemblance of dietary intakes between children of the family have reported this resemblance under the general title of siblings and have not investigated different sibling dyads, including brother-brother, sister-sister, and brother-sister, separately.

To our knowledge, there is no overall agreement between studies for the heritability of dietary intakes nor their resemblance among parents and Childs. Also, as mentioned earlier, many questions in the field of heritability have not been answered, or limited studies are available in the current literature. Therefore, in the present study, we intended to investigate the heritability of food intake, including different nutrients, minerals, and electrolytes, as well as the resemblance of dietary intakes in different age-sex dyads of the family in the Tehran Cardiometabolic Genetic Study (TCGS). This study's results can fill many scientific gaps regarding the role of genetics in food intake.

## Methods and materials

### Study participants and genealogy data

A prospective cohort study was made among families living in District 13 of Tehran to follow up on health-related traits every three years and record each outcome annually named TCGS [18]. Follow up have been held between 1998 and 2022, and nutrition habits were gathered every three years.

The TCGS participants were assessed for familial relationships, genetic data, dietary intakes, anthropometric parameters, biochemical factors, etc. For the present study, among the participants with complete dietary data for the third (*n* = 3568), fourth (*n* = 7956), fifth (7052), and sixth (*n* = 7720) survey of the TCGS, in each survey, participants aged ≥ 18 years were selected if who were not a pregnant and lactating woman, haven't the history of cancer, myocardial infarction, cardiovascular accident, and their nutritional data were measured for the first time. Of the total of 10,468 included participants (third survey (*n* = 2944, 28.12%), fourth survey (*n* = 4928, 47.07%), fifth survey (1605, 15.33%), and sixth survey (*n* = 991, 9.46%)), 668 subjects were excluded for their under, or over-reporting of energy intake (lower than 800 and higher than 4200 kcal/d) and 9798 participants remained for the data analyses. As mentioned above, 75.19% and 91.54% of participants' dietary data were collected in 3- and 6-year intervals.

Among these 9798 participants, 4338 families, including spouses, first-degree relative pairs (11,576 Parents/offspring and 3039 siblings), and second-degree relative pairs (including 4914 grandparents, avuncular, half-siblings, and cousins) were identified.

### Ethics declaration and consent form

This research was related to the Ph.D. thesis and was authorized by the local research ethics committee of the Endocrine Research Institute at the University of Shahid Beheshti Medical Science and the ethics committee of the Iran University of Medical Science (Research Ethical Code: IR.IUMS.REC.1401.283 and project number:22083). For their involvement in the survey, all subjects provided written informed consent. This study was carried out following the Helsinki Declaration.

### Dietary assessments

Dietary intakes of the study population were assessed using a valid and reliable semi-quantitative 168-item food frequency questionnaire (FFQ) [19, 20]. In each survey, trained dieticians, in a face-to-face interview, asked subjects to report their consumption frequency of each food item as portion sizes in household measures during the previous year, as daily, weekly, or monthly. Food frequencies are entered into an Excel sheet and then transformed into a grams scale. Data on participants' dietary intake were collected four times, including surveys third to the sixth. Generally, 33.5%, 33.5%, 25.5%, and 7.5% of participants had one, two, three, and four measurements. Nutrient intake for each participant was calculated as the average of his/her measurements (sum of measures divided by number of time of measurements).

### Nutrients intake assessment

We calculated the daily intake of energy, macronutrients (as both g/day and % of kcal), including carbohydrates, protein, fat, SFAs, MUFAs, PUFAs, trans fatty acids (TFAs), and also cholesterol, fiber, caffeine, mineral and electrolytes including calcium, phosphor, iron, zinc, copper, magnesium, manganese, chromium, selenium, sodium, potassium, and vitamins including vitamin A, D, E, K, thiamine (B1), riboflavin (B2), niacin (B3), pantothenic acid (B5), pyridoxine (B6), folate (B9), cobalamin (B12), and vitamin C all reported as dietary intake per 1000 kcal.

The energy contents of consumed foods were calculated using the United States Department of Agriculture (USDA) food composition table (FCT) (available at https://fdc.nal.usda.gov/fdc-app.html). Furthermore, the Iranian FCT was used to analysis of the local food items unavailable in the USDA FCT.

### Statistical analysis

Dietary nutrient intakes for each participant were adjusted by age and normalized for all analyses, as the normalized scores were calculated using the rank-based inverse normal transformation (INT) method [21]. Pedigree information was obtained using the S.A.G.E. software.

### Familial aggregation and spousal resemblance

The intraclass correlation coefficient (ICC) is commonly used to quantify the degree to which individuals with a fixed degree of relatedness (e.g. sister-sister, sister-brother, brother-brother, child-parent, etc.) resemble each other in terms of a quantitative trait. FCOR command of the S.A.G.E. software was used to estimate the ICC of all relative pairs to verify the family resemblance of dietary nutrient intakes for all relative pairs and spouses. Only significant ICCs are reported (*p* < 0.05).

### Family-based heritability

Family-based heritability indicated with h^2^ and its standard error (SE) ranges between 0 and 1 and measures the proportion of the phenotypic variance of the traits that appear in families for many reasons in addition to genetics, such as similarities in lifestyle and environment.

Classical likelihood-based was used to assess the family-based heritability of dietary nutrient traits. For each individual, there were fixed factors, including age and gender. We have used a Gaussian random-effects model with a covariance structure. Then, we included a random effect, k = N (0, K ${\sigma }_{g}^{2}$), where K is a kinship matrix, and ${\sigma }_{g}^{2}$ is the genomic variance. The response vector ${\varvec{y}}={\{y}_{i}\}$ was defined as the food groups' level for the i^th^ individual. The probit link was implemented $\mathrm{P}\left({y}_{i}=1|{G}_{i}\right)=\Phi \left({\eta }_{i}\right)$ where Φ is the cumulative distribution function (CDF) and η_i_ is a linear predictor given by:

$${\eta }_{i}=\mu +\sum_{k=1}^{q}{x}_{ik}{\beta }_{ik}+{g}_{i}$$

where μ is an intercept, x_ij_ is the k^th^ fixed factors, β_ij_ is the effects associated with the k^th^ fixed factors, and k_i_ is a total genetic effect of the i^th^ individual. The heritability was calculated at the level of the entire family, not for specific family pairs.

## Results

Pedigree and relative pairs information are provided in Table 1. There were 4338 families with a mean family size of 3.20 ± 2.89, including 1 to 32 members (2567 constituent pedigrees and 1572 singletons), 3627 sibships (1.60 ± 0.85; min = 1, max = 6). There were 14,615 first-degree relatives (11,576 Parents/offspring and 3039 siblings) and 4914 s-degree relatives, including grandparents, avuncular, half-siblings, and cousins.

**Table 1:** Relative-pairs information of the study participants

**Pairs type**			**Number of pairs**
**First-degree relative**	Parents/offspring		11,576
	Siblings (3627 pairs)	sister-sister	866
		brother-brother	735
		brother-sister	1438
**Second-degree relative**	Grandparents		3552
	Avuncular		937
	Half sibling		65
	Cousin		360

Demographic characteristics, nutrient intakes, and the mean and SE of heritability of nutrient intakes are shown in Table 2. Participants' mean ± SD of age and BMI (body mass index) were 42.0 ± 15.2 years and 27.2 ± 5.0 kg/m^2^, and 44.5% were males. Generally, participants consumed their energy intake by 56.6%, 29.4%, and 14.0% from carbohydrate, fat, and protein, respectively. MUFAs, SFAs, and then PUFAs are the main sources of fat intake, and it seems that study participants consume appropriate amounts of vitamins and minerals. The heritability of nutrients ranged from 3 to 21%, and the highest heritability was observed for protein (g/day) (21.1%), MUFAs (g/day) (21.1%), total fat (g/day) (20.6%), vitamin C (mg/1000 kcal) (20.4%), PUFAs (g/day) (20.0%), vitamin D (mcg/1000 kcal) (19.6%), carbohydrate (g/day) (19.1%), and energy intake (kcal/day) (18.6%), respectively. However, the lowest heritability was reported for calcium (mg/1000 kcal) (3.6%), iron (mg/1000 kcal) (6.3%), chromium (mg/1000 kcal) (6.5%), TFAs (g/day) (6.8%), and TFAs (% of Kcal) (6.9%). When calculated as the percent of total energy intake, Macronutrients had higher heritability than their net consumption amounts in grams. Among the minerals and electrolytes, copper and magnesium (each 14.2%) had the highest heritability, whereas calcium (3.6%), chromium (6.5%), potassium (9.2%), and sodium (9.4%) showed the lowest heritability, respectively. Among the vitamins, the highest heritability was related to vitamins C (20.4%), D (19.6%), B12 (15.5%), A (14.8%), and pantothenic acid (14.4%); however, the lowest was related to riboflavin (9.0%), vitamins E and K (each 9.8%), respectively.

**Table 2:** Demographic characteristics, dietary intakes, and heritability values of study participants

Variables^a^		≥ 18 years (*n* = 9798)		
		Mean ± SD or Median (Q1-Q3)	h^2b^ (%)	SE^c^(%)
**Age (y)**		42.0 ± 15.2	-	-
**Male (%)**		44.5	-	-
**BMI (kg/m**^**2**^**)**		27.2 ± 5.0	-	-
**Nutrients**				
**Macronutrients**	Energy intake (Kcal/d)	2411 ± 838	18.6	0.1
	Carbohydrate (% of Kcal)	56.6 ± 6.3	16.3	3.5
	*Carbohydrate (g/d)*	341 ± 116	19.1	0.1
	Protein (% of Kcal)	14.0 ± 2.6	15.4	3.5
	*Protein (g/d)*	84.6 ± 38.3	21.1	0.1
	Fat (% of Kcal)	29.4 ± 6.3	15.0	3.5
	*Fat (g/d)*	78.7 ± 38.0	20.6	0.1
	PUFAs (% of Kcal)	6.0 ± 2.1	15.4	3.5
	*PUFAs (g/d)*	14.7 (10.5 – 19.6)	20.0	3.4
	MUFAs (% of Kcal)	10.0 ± 2.6	12.6	3.6
	*MUFAs (g/d)*	24.7 (18.7 – 32.2)	21.1	0.1
	SFAs (% of Kcal)	9.6 ± 3.2	16.5	3.5
	*SFAs (g/d)*	23.6 (17.8 – 31.1)	9.4	3.6
	TFAs (% of Kcal)	0.00 (0.00—0.00)	6.9	3.7
	*TFAs (g/d)*	0.00 (0.00—0.00)	6.8	3.7
	Cholesterol (mg/1000 kcal)	95.2 ± 40.3	13.5	3.5
	Fiber (g/1000 kcal)	18.6 ± 6.7	12.8	3.6
	Caffeine (mg/1000 kcal)	44.7 (25.5 – 74.2)	13.3	3.5
**Minerals and electrolytes**	Calcium (mg/1000 kcal)	591 ± 188	3.6	3.8
	Phosphor (mg/1000 kcal)	678 ± 121	11.2	3.6
	Iron (mg/1000 kcal)	15.4 ± 8.9	6.3	3.7
	Zinc (mg/1000 kcal)	5.3 (4.7 – 5.9)	12.0	3.6
	Copper (mg/1000 kcal)	0.76 ± 0.33	14.2	3.5
	Magnesium (mg/1000 kcal)	189 ± 41	14.2	3.5
	Manganese (mg/1000 kcal)	3.6 (2.8 – 4.6)	10.7	3.6
	Chromium (mg/1000 kcal)	0.04 (0.02 –0.07)	6.5	3.7
	Selenium (mcg/1000 kcal)	52.5 ± 13.7	11.8	3.6
	Sodium (mg/1000 kcal)	1659 ± 747	9.4	3.6
	Potassium (mg/1000 kcal)	1860 ± 512	9.2	3.6
**Vitamins**	Vitamin A (mcg/1000 kcal)	255 ± 123	14.8	3.5
	Vitamin D (mcg/1000 kcal)	0.71 (0.36 – 1.20)	19.6	3.4
	Vitamin E (mg/1000 kcal)	5.8 ± 1.8	9.8	3.6
	Vitamin K (mcg/1000 kcal)	112 (62—192)	9.8	3.6
	Thiamine (mg/1000 kcal)	0.87 ± 0.17	10.9	3.6
	Riboflavin (mg/1000 kcal)	0.94 ± 0.29	9.0	3.6
	Niacin (mg/1000 kcal)	8.8 ± 1.8	10.9	3.6
	Pantothenic acid (mg/1000 kcal)	2.5 ± 0.46	14.4	3.4
	Pyridoxine (mg/1000 kcal)	0.91 ± 0.35	13.8	3.5
	Folate (mcg/1000 kcal)	247 ± 43	13.5	3.5
	Vitamin B12 (mcg/1000 kcal)	1.7 ± 0.9	15.5	3.5
	Vitamin C (mg/1000 kcal)	70.6 ± 38.0	20.4	3.4

*Abbreviations*: *PUFAs* polyunsaturated fatty acids, *MUFAs* monounsaturated fatty acids, *SFAs* saturated fatty acids, *TFAs* trans fatty acids

^a^Data were represented as mean ± SD, or median (IQR 25–75) for continuous variables and percent for categorical variables

^b^Heritability of nutrient intakes determined by using the Gaussian random-effects model with a covariance structure calculated as a percentage (range 0–100%)

^c^Standard error of heritability of nutrient intakes calculated as a percentage (range 0–100%)

**Fig. 1:**
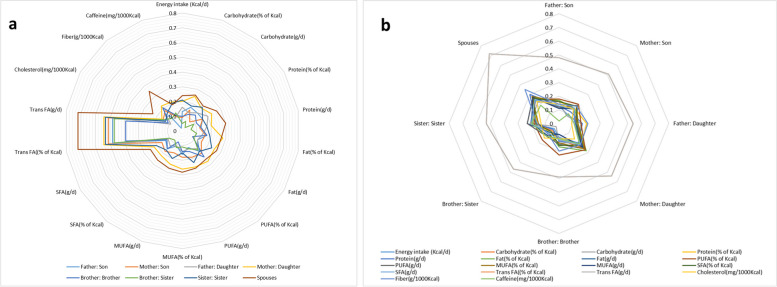
The correlation of each macronutrient intake among the adult (≥ 19 years) familial pairs participated (**a**) and the correlation of the macronutrient intake in each familial pair (**b**) in the Tehran lipid and glucose cohort study

The correlation of each macronutrient intake among the adult (≥ 19 years) familial pairs who participated in the Tehran lipid and glucose cohort study is shown in Fig. 1a-b and Supplementary Table 1. Except for the intake of total fat (% of Kcal), PUFAs (% of Kcal), and caffeine, the highest correlation of macronutrient intake was seen between spouses. Intake of total fat (% of Kcal), PUFAs (% of Kcal), and caffeine in mother-daughter pairs showed the highest correlation. Intake of TFAs (as g/day and % of Kcal) showed the highest correlation among all nutrients and in each dyad. The lowest correlation of TFAs intake was seen in brother-brother pairs. The lowest correlation among all macronutrients was for carbohydrate intake (% of Kcal) in brother-sister dyads.

**Fig. 2:**
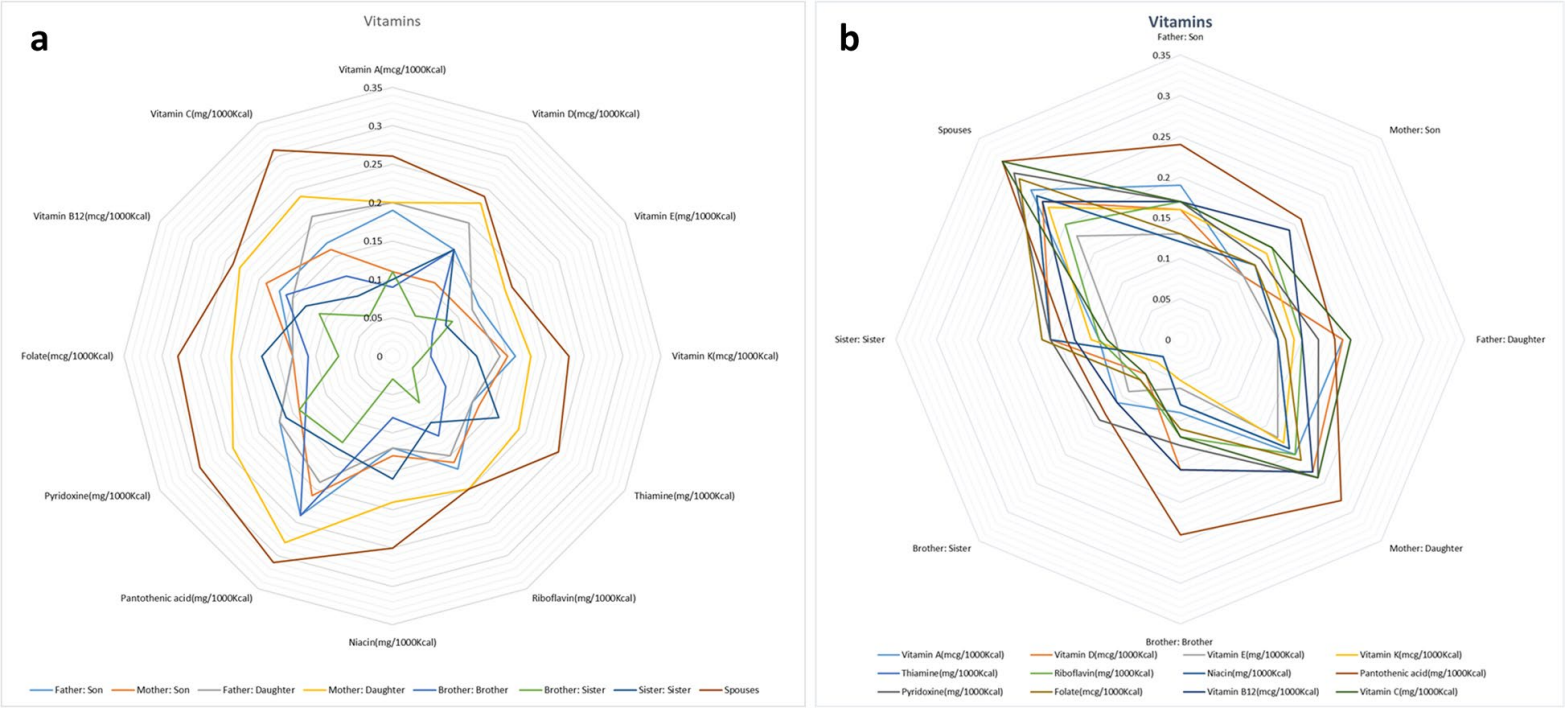
The correlation of each vitamin intake among the adult (≥ 19 years) familial pairs participated (**a**) and the correlation of the vitamins’ intake in each familial pair (**b**) in the Tehran lipid and glucose cohort study

The correlation of each vitamin intake between the adult (≥ 19 years) familial pairs who participated in the Tehran lipid and glucose cohort study is shown in Fig. 2a-b and Supplementary Table 2. Vitamin C and pantothenic acid had the highest correlation among spouses. In all pairs, except father-daughter, brother-sister, and sister-sister, the highest correlation was related to the intake of pantothenic acid. The lowest correlation was related to the vitamin D intake in brother-brother pairs. The highest correlation in the intake of vitamins was observed among spouses. Except for pyridoxine, vitamin A, and vitamin E, the lowest correlation was seen in the intake of other vitamins among brother-sister pairs.

**Fig. 3:**
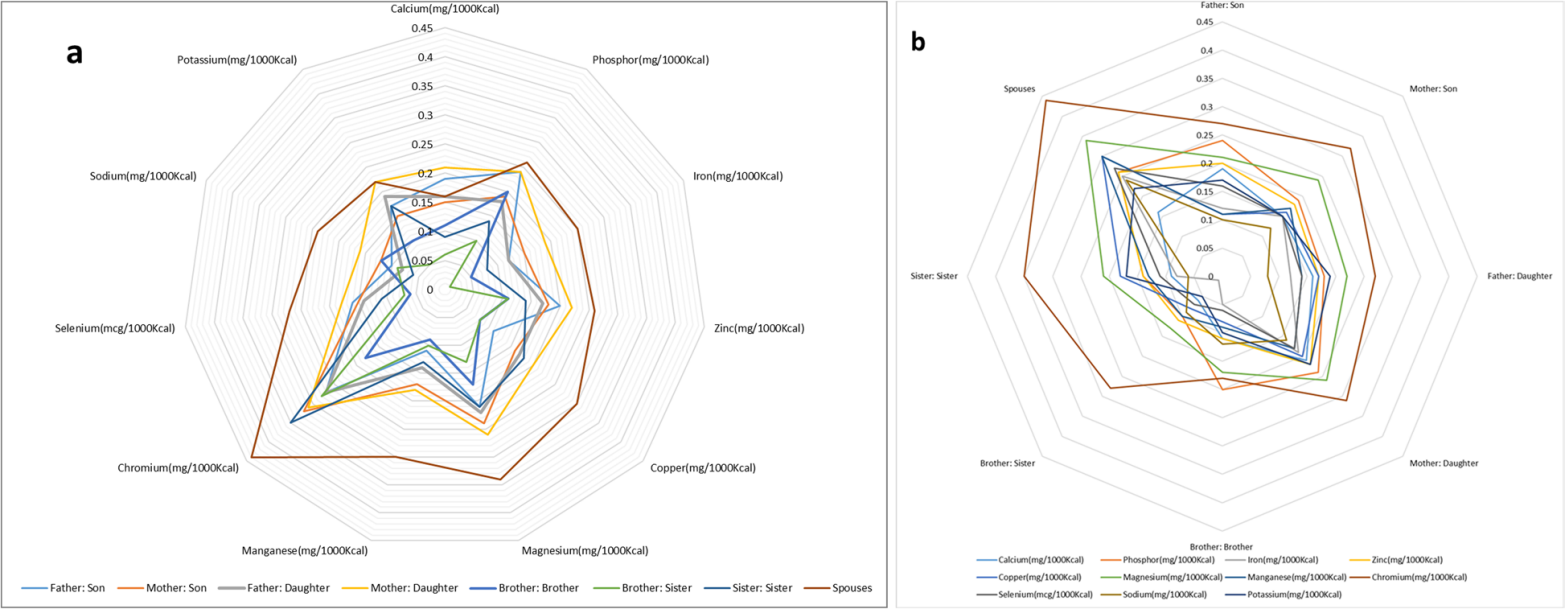
The correlation of each mineral or electrolyte intake among the adult (≥ 19 years) familial pairs participated (**a**) and the correlation of the minerals or electrolytes’ intake in each familial pair (**b**) in the Tehran lipid and glucose cohort study

The correlation of each mineral or electrolyte intake between the adult (≥ 19 years) familial pairs who participated in the Tehran lipid and glucose cohort study is shown in Fig. 3a-b and Supplementary Table 3. The highest correlation between minerals and electrolytes in all pairs was related to chromium intake. Spouses had the highest correlation in receiving all minerals and electrolytes except calcium. Brother-sister pairs had the lowest correlation in intake of all minerals and electrolytes except chromium, selenium, and sodium. In other pairs, observed correlations were generally between spouses and brother-sister pairs. Chromium intake showed the highest correlation among all pairs except brother-brother. The highest correlation in chromium intake was seen among spouses, and the lowest was in brother-brother pairs. Sodium intake showed the lowest correlation in all pairs except brother-brother, brother-sister, and spouses.

## Discussion

In the present study, we investigated the familial resemblance of dietary intakes, including energy intake, macronutrients, and micronutrients, between parents and their children (aged ≥ 19 years old) based on data from the TCGS study in Iran. We also assessed the Iranian population's family-based heritability of energy and nutrient intake. The results of our study showed that the degree of resemblance of energy intake and most nutrients between spouses or between parents and children is weak to moderate; however, for some food components, such as TFAs, pantothenic acid, and chromium, the high resemblance of intake is observed especially among spouses. The findings of our study indicated that the rate of resemblance in intakes of nutrients and energy between different pairs of families could be different, which indicates the influence of the environment and family in food choices between family members or possible parental effects on offspring's nutrient intakes or their food habits.

Based on the dietary reference intakes, including Recommended Dietary Allowances (RDA) or Adequate Intakes (AI), the intakes of the participants in this study for macronutrients and their components, fiber, minerals, and most of the vitamins were acceptable, and in most cases higher than the minimum daily recommended level [22]. It should be noted we used the USDA FCT to estimate nutrient intake in the participants, which has caused an overestimation of daily nutrient intake in individuals. This overestimation, especially for fiber and micronutrients, is clearly visible based on the results of Table 1. For instance, according to the findings of this study, the intake of calcium was more than the RDA for individuals, however, in reality, the consumption of dairy products as the main source of calcium is low among the Iranian population due to the high cost [23–25]. Therefore, low intake of dietary calcium is almost common in the Iranian adult population, which is hidden from our view in this study because of overestimation.

The intake of participants for some vitamins, including vitamins A and D was observed less than the minimum daily requirement. In the current study, participants' intake for vitamin D was 0.71 mcg /1000kcal per day, which is far from the RDA for adults (15 mcg per day); this finding was expected because the food sources with high vitamin D content (such as fish oil, calf liver, cheese, and egg yolk) or vitamin D-fortified foods have a low consumption among the Iranian population [26]. Also, the high prevalence of vitamin D deficiency in Iranian adults confirms the low intake of vitamin D food sources or less exposure to sunlight in them [26, 27]. The intake of vitamin A (255 mcg/1000 kcal/day) in study participants is somewhat less than the RDA (700–900 mcg/day), which is expected due to the low consumption of high vitamin A content foods in the Iranian population, however, this rarely causes an obvious deficiency of this vitamin and the occurrence of its complications in the Iranian population.

In the current study, we showed a wide variation in the degree of resemblance in the intake of nutrients between spouses, parents–children, and siblings (r: less than 0.1 to more than 0.7), that in an epidemiological study with a high sample size, these results are expected and reasonable. Also, according to our study's results, the most resemblance in the intake of nutrients and calories based on different family pairs has been observed among spouses and then between parents—offspring (especially mother-daughter). The lowest similarity in food intake is also among siblings. Our findings are comparable with the evidence from previous studies [16, 28–31]. Studies on the Tehranian population suggested a weak to moderate correlation between nutrient intakes of parent–offspring dyads that lived with their parents, and this correlation for the nutrient intakes of younger and older generations with parents was very lower and non-significant [29]. Also, the results of a study on the TCGS population revealed that the correlation reported for food consumption and dietary habit in father-offspring dyads were lower than in mother–offspring dyads [16]. Based on Robinson et al. Study, a possible moderate-to-strong dietary correlation between healthy spouses was observed; also, they suggested that similarities in mother–offspring dietary intakes were slightly higher than father–children intake [28]. A study on the USA population reported a weak resemblance in parent–child dietary intakes. They suggested that the resemblance can be differed based on types of nutrients and food groups, types of parent–child dyads, and the characteristics of population groups such as age, ethnicity, and socio-economic status [32]. Furthermore, Shrivastava et al. study has shown a moderate positive correlation between parents-child in dietary intake and suggested that dietary resemblances in mother–offspring are stronger than dietary resemblances of father–offspring [30]. A cohort study conducted on the Australian population reported a weak mother–adult offspring dietary resemblance. They declared that dietary resemblance in parent–offspring can differ based on the type of nutrients, offspring's gender, and living arrangements. In the study mentioned above, dietary resemblance in mother-daughter dyads was slightly greater than in mother-son dyads [31]. Even more findings from an investigation on a sample of Finnish preschoolers and their parents reported that mother–child resemblance was higher than father-child resemblance, which indicates a parent-respondent interaction [33].

In general, the results of our study, as well as the previous evidence, showed that the resemblance in nutrients intake based on various familial pairs is somewhat variable; these possible differences in familial resemblance in nutrients intakes may be affected by heritability, similarity in the environment, culture, and social conditions of between family members living in the same household, and social homogamy between spouses. In the Iranian population, parents may have more meals together than their children over a long period of a year. Also, offspring may have different food choices than their parents, including snacks and drinks, depending on their environments during the day. Furthermore, young individuals' dietary patterns and food preferences are influenced by various complex factors. In a comprehensive view, along with the family environment as an important factor, other factors, including social and biological factors, are key determinants of food choices that influence on nutrient intake and diet quality of individuals [34].

Consistent with the results of some previous studies [29, 30, 32, 35], our findings showed that for most nutrients, there is a greater resemblance between the mothers -daughters in comparison to other parent–offspring pairs. Although it is not completely known why girls had higher correlations with their parents (especially with mothers) than sons; however this difference in the correlation coefficient can be due to some possible reasons. In Iranian society, mothers and girls spend more time at home and are involved more time in cooking and preparing meals compared to fathers and sons, which can be effective in increasing the similarity of nutrient intake between them [29], while sons spend most of their time outside the house with friends or colleagues than with their families during the day that this behavior has also been observed in boys from other community [35]. It has been suggested that peer group effects can be higher than familial influences on sons, which caused a lower correlation between boys' nutrients intake and their parents [35]. Also, the biological, physiological, psychosocial, and behavioral differences between girls and boys can be one of these reasons. Considering that the stages of physical growth, maturity, and the period of body development of boys and girls happen in different age periods based on biological differences, so, the dietary requirements of boys and girls can be different based on the effects of these factors in the age period that subsequently lead differences in food choices between them during the adult life period. e.g., at a certain age, boys are in early youth regarding physical growth and development; however, girls are fully mature. Moreover, physiological differences in boys and girls can also play a role in the difference in nutrients resemblance of parents- offspring; due to the difference in body parts and physical strength, boys can be more physically active than girls. Therefore, the effects of the environment, parents, friends, and peers on nutritional intake and food habits can be different between boys and girls [36].

Among different nutrients, the highest correlation between familial pairs is for the intakes of TFAs, chromium, and pantothenic acid. Several reasons can explain these findings. For example, in Iranian society, nuts, which can contain a high amount of chromium and some vitamins such as pantothenic acid, are consumed on special days (such as celebrations, parties, Nowruz, etc.) when all family members gather together. Also, on some days of the month, family members have meals together outside the home, including in restaurants that probably have harmful food choices such as pizza, hamburger, or other fast food choices; these meals have caused family members to have a similar intake of harmful food compounds such as TFAs.

In the present study, we computed the possible family-based heritability of energy, macronutrients, and micronutrients, which indicates a weak to moderate heritability of them (h^2^: 3.6% for calcium and 21.1% for protein). Based on our results, the highest heritability was seen for macronutrients and some vitamins such as protein (21.1%), MUFAs (21.1%), total fats (20.6%), vitamin C (20.4%), PUFAs (20.0%), vitamin D (19.6%), carbohydrate (19.1%), and energy intake (18.6%), respectively. In contrast, for some minerals such as calcium (3.6%), iron (6.3%), and chromium (6.5%), the lowest familial-based heritability was observed. One of the reasons for the high correlation of macronutrients and energy between family pairs compared to micronutrients could be that FFQ, as the main nutritional tool in epidemiological studies, provides a high estimate of macronutrients and energy intake in individuals and can present good measures of these intakes, while the estimation of micronutrient intake, such as trace elements, may be low or far from the actual intake based on the data obtained from FFQ [20, 37]. Also, our results show that consuming foods containing macronutrients and high energy in Iranian society in traditional food patterns is more heritable. Considering that In Iranian families, the consumption of meals containing a high amount of refined grains (various types of bread, rice, and macaroni, etc.), legumes, and types of different meats are done mostly simultaneously and jointly by family members and so all family members who live in the same home have close consumption of these food groups, therefore, we have observed a high familial based heritability for carbohydrates, fats, protein, and energy intake. Also, in Iranian families, fruits are usually consumed when all the family members are at home at the same time; this eating behavior has caused us to observe a high familial heritability for intakes of carbohydrates and vitamin C for the Iranian population in the present study. However, the consumption of vegetables, nuts, and dairy products, which contain high amounts of some vitamins and minerals, is mostly not done jointly and at the same time with other family members in Iranian societies, and their intake may vary among family members; therefore, a lower familial heritability has been reported for most of minerals and vitamins, such as calcium, iron, chromium, riboflavin, and Vitamin K in this present study.

The several strengths of our study deserve to mention. The present study is the first to examine the possible nutrient intake resemblance in various familial pairs, including parent pairs, parent-offspring, and siblings (aged ≥ 19 years old). Also, as the first study, we conducted the family-based heritability for various nutrients in individuals aged ≥ 19 years old. Also, in the present study, we focused on the parent and offspring nutrient intakes, separately by sex, which indicates another dimension of perceptions of familial resemblance and family-based heritability. We are also the first study that determined familial resemblance and heritability for all nutrients (macronutrients and micronutrients) and energy intake. Furthermore, we collected dietary data of parents-offspring pairs using a valid and reliable FFQ, which minimized the potential measurement error. However, our study has some limitations; we did not check the resemblance of nutrient intakes for parent-young offspring dyads that lived with their parents and offspring not living at home with their parents. Also, no data are available on the number of shared meals of parent–offspring dyads that should be controlled. There is no data on the number of meals family members spent together outside the home, such as in restaurants. The lack of data on the unmeasured variables, such as the socioeconomic status of the subjects, which can significantly influence food choices and nutrient intake is another limitation of the current study.

## Conclusions

In conclusion, the current population-based study revealed that the resemblance of nutrient intake among spouses was greater than in parent-offspring. This high resemblance was shown in both healthy and harmful dietary components. The strongest correlation between spouses was observed for TFAs, chromium, fiber, pantothenic acid, and vitamin C. Also, weak-to-moderate similarities were observed between the nutrient intakes of parents and offspring; the highest correlation between parents and offspring was reported for TFAs, total fat, PUFAs, and MUFAs. The similarity in parent–offspring nutrient intake was different, and the correlation in mother-girls nutrient intakes was mostly greater than other parent–child correlations.

The findings of the current study suggested that an individual's nutrient intake could somewhat be influenced by genetics, family relationships, and the effects of parents, although the significant influence of environmental factors should not be ignored. Recommending to increase the family consumption of food choices containing nutrients that had a high correlation between pairs of families, especially between parents and children, can increase the intake of these micronutrients simultaneously in all family members. However, the low correlation of some other nutrients between pairs of families indicates the influence of other environmental factors on people's food intake, such as the influence of peers, work environment, etc.; it is suggested that changes in people's meals outside the home should be considered to increase the intake of these nutrients. Further studies with longitudinal design and high sample size are needed to investigate the family-based heritability and the intergenerational dietary effects in other populations.

### Supplementary Information

**Additional file 1: Supplementary Table 1. **The correlation coefficient of dietary intake of macronutrients among the adult (≥19 year) familial pairs participated in the Tehran lipid and glucose cohort study.

**Additional file 2: Supplementary Table 2. **The correlation coefficient of dietary intake of vitamins among the adult (≥19 year) familial pairs participated in the Tehran lipid and glucose cohort study.

**Additional file 3: Supplementary Table 3. **The correlation coefficient of dietary intake of minerals and electrolytes among the adult (≥19 year) familial pairs participated in the Tehran lipid and glucose cohort study.

## Data Availability

The datasets analyzed in the current study are available from the corresponding author on reasonable request.

## Abbreviation

BMI: Body mass index
TCGS: Tehran Cardiometabolic Genetic Study
FFQ: Food frequency questionnaire
SFAs: Saturated fatty acids
MUFAs: Monounsaturated fatty acids
PUFAs: Polyunsaturated fatty acids
USDA: United States Department of Agriculture
FCT: Food composition table
ICC: Intraclass correlation
TFAs: Trans fatty acids
