# Supplementary material for: Familial resemblance and family-based heritability of nutrients intake in Iranian population: Tehran cardiometabolic genetic study
Source: BMC Public Health. 2023 Sep 14;23:1789. doi: 10.1186/s12889-023-16708-2 (PMC10500786; doi:10.1186/s12889-023-16708-2)
Supplement: Supplementary file 1 — Additional file 1: Supplementary Table 1. The correlation coefficient of dietary intake of macronutrients among the adult (≥19 year) familial pairs participated in the Tehran lipid and glucose cohort study. [file 12889_2023_16708_MOESM1_ESM.docx]

**Supplementary Table 1.** The correlation coefficient of dietary intake of macronutrients among the adult (≥19 year) familial pairs participated in the Tehran lipid and glucose cohort study

| **Macronutrients intake** | **Father: Son** | | **Mother: Son** | | **Father: Daughter** | | **Mother: Daughter** | | **Brother: Brother** | | **Brother: Sister** | | **Sister: Sister** | | **Spouses** | |
| --- | --- | --- | --- | --- | --- | --- | --- | --- | --- | --- | --- | --- | --- | --- | --- | --- |
|  | **r** | **SE** | **r** | **SE** | **r** | **SE** | **r** | **SE** | **r** | **SE** | **r** | **SE** | **r** | **SE** | **r** | **SE** |
| Energy intake (Kcal/d) | 0.13*** | 0.03 | 0.09*** | 0.02 | 0.16*** | 0.03 | 0.20*** | 0.02 | 0.10** | 0.03 | 0.05 | 0.02 | 0.21*** | 0.03 | 0.24*** | 0.02 |
| Carbohydrate(% of Kcal) | 0.17*** | 0.02 | 0.15*** | 0.02 | 0.13*** | 0.03 | 0.25*** | 0.02 | 0.12*** | 0.03 | 0.07** | 0.02 | 0.18*** | 0.03 | 0.26*** | 0.02 |
| *Carbohydrate(g/d)* | 0.16*** | 0.02 | 0.08** | 0.02 | 0.16*** | 0.03 | 0.20*** | 0.02 | 0.14*** | 0.03 | 0.03 | 0.02 | 0.21*** | 0.03 | 0.22*** | 0.02 |
| Protein(% of Kcal) | 0.16*** | 0.02 | 0.16*** | 0.02 | 0.20*** | 0.02 | 0.23*** | 0.02 | 0.11** | 0.03 | 0.09** | 0.03 | 0.16*** | 0.03 | 0.27*** | 0.02 |
| *Protein(g/d)* | 0.13*** | 0.03 | 0.12*** | 0.02 | 0.17*** | 0.02 | 0.20*** | 0.02 | 0.13*** | 0.03 | 0.06* | 0.02 | 0.17*** | 0.03 | 0.30*** | 0.02 |
| Fat(% of Kcal) | 0.17*** | 0.03 | 0.16*** | 0.02 | 0.14*** | 0.03 | 0.28*** | 0.02 | 0.17*** | 0.03 | 0.10*** | 0.02 | 0.19*** | 0.03 | 0.27*** | 0.02 |
| *Fat(g/d)* | 0.12*** | 0.03 | 0.15*** | 0.02 | 0.12*** | 0.03 | 0.25*** | 0.02 | 0.08* | 0.03 | 0.09*** | 0.02 | 0.23*** | 0.03 | 0.27*** | 0.02 |
| PUFAs(% of Kcal) | 0.18*** | 0.03 | 0.20*** | 0.02 | 0.16*** | 0.03 | 0.27*** | 0.02 | 0.23*** | 0.03 | 0.17*** | 0.02 | 0.18*** | 0.03 | 0.25*** | 0.02 |
| *PUFAs(g/d)* | 0.12*** | 0.03 | 0.19*** | 0.02 | 0.13*** | 0.03 | 0.26*** | 0.02 | 0.15*** | 0.03 | 0.13*** | 0.02 | 0.23*** | 0.03 | 0.27*** | 0.02 |
| MUFAs(% of Kcal) | 0.16*** | 0.03 | 0.18*** | 0.02 | 0.14*** | 0.02 | 0.26*** | 0.02 | 0.14*** | 0.03 | 0.11*** | 0.02 | 0.15*** | 0.03 | 0.28*** | 0.02 |
| *MUFAs(g/d)* | 0.11*** | 0.03 | 0.16*** | 0.02 | 0.11*** | 0.03 | 0.24*** | 0.02 | 0.08* | 0.03 | 0.12*** | 0.02 | 0.20*** | 0.03 | 0.27*** | 0.02 |
| SFAs(% of Kcal) | 0.16*** | 0.03 | 0.18*** | 0.02 | 0.13*** | 0.03 | 0.22*** | 0.02 | 0.16*** | 0.03 | 0.08** | 0.02 | 0.17*** | 0.03 | 0.26*** | 0.02 |
| *SFAs(g/d)* | 0.13*** | 0.03 | 0.16*** | 0.02 | 0.11*** | 0.03 | 0.22*** | 0.02 | 0.12** | 0.03 | 0.10*** | 0.02 | 0.20*** | 0.03 | 0.25*** | 0.02 |
| TFAs((% of Kcal) | 0.48*** | 0.02 | 0.51*** | 0.01 | 0.54*** | 0.02 | 0.54*** | 0.01 | 0.39*** | 0.03 | 0.47*** | 0.02 | 0.53*** | 0.03 | 0.72*** | 0.01 |
| *TFAs(g/d)* | 0.48*** | 0.02 | 0.51*** | 0.02 | 0.54*** | 0.02 | 0.54*** | 0.01 | 0.39*** | 0.03 | 0.47*** | 0.02 | 0.53*** | 0.03 | 0.72*** | 0.01 |
| Cholesterol(mg/1000Kcal) | 0.14*** | 0.03 | 0.15*** | 0.02 | 0.09** | 0.03 | 0.16*** | 0.02 | 0.11* | 0.04 | 0.15*** | 0.02 | 0.13*** | 0.03 | 0.23*** | 0.02 |
| Fiber(g/1000Kcal) | 0.14*** | 0.03 | 0.18*** | 0.02 | 0.21*** | 0.03 | 0.22*** | 0.02 | 0.20*** | 0.03 | 0.11*** | 0.03 | 0.16*** | 0.03 | 0.35*** | 0.02 |
| Caffeine(mg/1000Kcal) | 0.02 | 0.03 | 0.10*** | 0.02 | 0.11*** | 0.03 | 0.21*** | 0.02 | 0.11** | 0.03 | 0.13*** | 0.03 | 0.20*** | 0.03 | 0.19*** | 0.03 |

*** P-value <0.001, ** P-value <0.01, * P-value <0.05

Abbreviations: PUFAs: polyunsaturated fatty acids, MUFAs: monounsaturated fatty acids, SFAs: saturated fatty acids, TFAs: trans fatty acids
