# Supplementary material for: Familial resemblance and family-based heritability of nutrients intake in Iranian population: Tehran cardiometabolic genetic study
Source: BMC Public Health. 2023 Sep 14;23:1789. doi: 10.1186/s12889-023-16708-2 (PMC10500786; doi:10.1186/s12889-023-16708-2)
Supplement: Supplementary file 2 — Additional file 2: Supplementary Table 2. The correlation coefficient of dietary intake of vitamins among the adult (≥19 year) familial pairs participated in the Tehran lipid and glucose cohort study. [file 12889_2023_16708_MOESM2_ESM.docx]

**Supplementary Table 2.** The correlation coefficient of dietary intake of vitamins among the adult (≥19 year) familial pairs participated in the Tehran lipid and glucose cohort study

| **Vitamins** | **Father: Son** | | **Mother: Son** | | **Father: Daughter** | | **Mother: Daughter** | | **Brother: Brother** | | **Brother: Sister** | | **Sister: Sister** | | **Spouses** | |
| --- | --- | --- | --- | --- | --- | --- | --- | --- | --- | --- | --- | --- | --- | --- | --- | --- |
|  | **r** | **SE** | **r** | **SE** | **r** | **SE** | **r** | **SE** | **r** | **SE** | **r** | **SE** | **r** | **SE** | **r** | **SE** |
| Vitamin A(mcg/1000Kcal) | 0.19*** | 0.02 | 0.11*** | 0.02 | 0.20*** | 0.03 | 0.20*** | 0.02 | 0.09* | 0.03 | 0.11*** | 0.03 | 0.10** | 0.03 | 0.26*** | 0.02 |
| Vitamin D(mcg/1000Kcal) | 0.16*** | 0.03 | 0.11 | 0.02 | 0.20*** | 0.02 | 0.23*** | 0.02 | 0.16*** | 0.03 | 0.06* | 0.02 | 0.16*** | 0.03 | 0.24*** | 0.02 |
| Vitamin E(mg/1000Kcal) | 0.13*** | 0.03 | 0.11*** | 0.02 | 0.12*** | 0.03 | 0.17*** | 0.02 | 0.06 | 0.04 | 0.09** | 0.03 | 0.08* | 0.03 | 0.18*** | 0.03 |
| Vitamin K(mcg/1000Kcal) | 0.16*** | 0.03 | 0.15*** | 0.02 | 0.14*** | 0.02 | 0.18*** | 0.02 | 0.05 | 0.03 | 0.04 | 0.03 | 0.11** | 0.04 | 0.23*** | 0.02 |
| Thiamine(mg/1000Kcal) | 0.12*** | 0.03 | 0.13*** | 0.02 | 0.12*** | 0.03 | 0.19*** | 0.02 | 0.08* | 0.03 | 0.03 | 0.03 | 0.16*** | 0.03 | 0.25*** | 0.02 |
| Riboflavin(mg/1000Kcal) | 0.17*** | 0.03 | 0.16*** | 0.02 | 0.15*** | 0.03 | 0.20*** | 0.02 | 0.12*** | 0.03 | 0.07* | 0.03 | 0.10** | 0.03 | 0.20*** | 0.03 |
| Niacin(mg/1000Kcal) | 0.12*** | 0.03 | 0.13*** | 0.02 | 0.12*** | 0.03 | 0.19*** | 0.02 | 0.08* | 0.03 | 0.03 | 0.03 | 0.16*** | 0.03 | 0.25*** | 0.02 |
| Pantothenic acid(mg/1000Kcal) | 0.24*** | 0.02 | 0.21*** | 0.02 | 0.19*** | 0.02 | 0.28*** | 0.02 | 0.24*** | 0.03 | 0.13*** | 0.03 | 0.14*** | 0.03 | 0.31*** | 0.02 |
| Pyridoxine(mg/1000Kcal) | 0.17*** | 0.02 | 0.14*** | 0.02 | 0.17*** | 0.03 | 0.24*** | 0.02 | 0.13*** | 0.03 | 0.14*** | 0.03 | 0.16*** | 0.03 | 0.29*** | 0.02 |
| Folate(mcg/1000Kcal) | 0.13*** | 0.02 | 0.13*** | 0.02 | 0.13*** | 0.03 | 0.21*** | 0.02 | 0.11** | 0.03 | 0.07* | 0.03 | 0.17*** | 0.03 | 0.28*** | 0.02 |
| Vitamin B12(mcg/1000Kcal) | 0.17*** | 0.02 | 0.19*** | 0.02 | 0.15*** | 0.02 | 0.23*** | 0.02 | 0.16*** | 0.03 | 0.11*** | 0.02 | 0.13*** | 0.03 | 0.24*** | 0.02 |
| Vitamin C(mg/1000Kcal) | 0.17*** | 0.03 | 0.16*** | 0.02 | 0.21*** | 0.02 | 0.24*** | 0.02 | 0.12*** | 0.03 | 0.06 | 0.03 | 0.09 | 0.03 | 0.31*** | 0.02 |

*** P-value <0.001, ** P-value <0.01, * P-value <0.05
